# Empirical study of correlated survival times for recurrent events with proportional hazards margins and the effect of correlation and censoring

**DOI:** 10.1186/1471-2288-13-95

**Published:** 2013-07-24

**Authors:** Rodrigo Villegas, Olga Julià, Jordi Ocaña

**Affiliations:** 1, Universitat de Barcelona, Barcelona, Spain; 2 Departament de Probabilitat, Lògica i Estadística, Universitat de Barcelona, Barcelona, Spain; 3 Departament d’Estadística, Universitat de Barcelona, Barcelona, Spain

## Abstract

**Background:**

In longitudinal studies where subjects experience recurrent incidents over a period of time, such as respiratory infections, fever or diarrhea, statistical methods are required to take into account the within-subject correlation.

**Methods:**

For repeated events data with censored failure, the independent increment (AG), marginal (WLW) and conditional (PWP) models are three multiple failure models that generalize Cox’s proportional hazard model. In this paper, we revise the efficiency, accuracy and robustness of all three models under simulated scenarios with varying degrees of within-subject correlation, censoring levels, maximum number of possible recurrences and sample size. We also study the methods performance on a real dataset from a cohort study with bronchial obstruction.

**Results:**

We find substantial differences between methods and there is not an optimal method. AG and PWP seem to be preferable to WLW for low correlation levels but the situation reverts for high correlations.

**Conclusions:**

All methods are stable in front of censoring, worsen with increasing recurrence levels and share a bias problem which, among other consequences, makes asymptotic normal confidence intervals not fully reliable, although they are well developed theoretically.

## Background

Cox’s proportional hazards model [1] is the most commonly used model for clinical trial data and provides reliable estimates of survival times, as well as the relative risk associated with time-to-event occurrence. As a semi-parametric model, it does not have any constraints on distributional assumptions, which makes it an attractive alternative to parametric models. However, it is not suitable for recurrent event data because survival times in the standard model terminate at the time of the event. In addition, trying to use multiple failure times is inappropriate since events within individuals may be correlated. In this case, the assumption of independence in the standard Cox regression model is violated, introducing statistical complications. To avoid the errors resulting from analyzing correlated repeated events, sometimes repeated events are disregarded and time to first event is only used, which is clearly a suboptimal approach. Multiple-event analytical techniques for survival data have been developed during the last decades [2] but, as a result of their complex structure and computational requirements, they have not been commonly applied. Advancement in statistical software in recent years has made these methods more accessible to researchers. As a consequence, their use has gradually increased in areas such as clinical research. Text books like Therneau and Grambsch, [2], describe these models in detail with examples, while research articles, such as that of Kelly and Lim [3], provide a very comprehensive explanation of their application to multiple-failure data, with extensive discussion about assumptions. There are three commonly used statistical methods for the analysis of recurrent events: Anderson-Gill model [4], Prentice-William-Peterson model [5] and Wei-Lin and Weissfeld model [6]. However, the benefits and drawbacks of each method are still unclear to the medical researchers, specially under varying strengths of correlation between survival times. The aim of this work is to study, through simulations and a real data set, the differences between the three models under presence of low to moderate correlation in survival times. With respect to similar studies like Kelly and Lim [3], we very approximately control the within individual correlation between consecutive recurrent events and we allow decreasing correlations as long as distance between recurrent events increases. In addition our simulation design takes care of different censoring levels and different numbers of recurrences, which are not fixed at 4 events as in [3]. We briefly describe the models, discuss in more detail the simulation procedure, and discuss the results of fitting the simulated data for the different models. In the Example section, we provide an illustration of the models performance using real data from a study in infants with respiratory illness recurrences.

## Methods

### Basic notations

It is common to model survival times through the hazard function. The Cox proportional hazard model for right censored survival data specifies the hazard function for the *i*^*th*^ individual by

(1) $$h_{i}(t)=h_{0}(t)\exp(\beta^{\prime }Z_{i}),$$

where *t* represents time, *Z*_*i*_ is the vector of covariates of the *i*^*th*^ individual, *β* is a vector of regression coefficients and *h*_0_(*t*) is the so-called baseline hazard function that corresponds to the hazard function for an individual with covariables set to zero. As model (1) is formulated through the hazard function, the simulation of appropriate survival times for this model is not straightforward, the effect of the covariates must be translated from the hazards to the survival time. We need to simulate the survival times since software packages for Cox models require the individual survival time data, not the hazard function. Suppose that there are *n* individuals and that each individual can experience *K* failures. Times of failure are subject to right censoring and we consider that censoring is non informative, which means that knowledge of a censoring time for an individual or subject provides no further information about the individual’s probability of survival at a future time. Let *T*_*ij*_ be the time when the *j*^*th*^ failure occurs for the *i*^*th*^ subject, measured from the subject’s study enrollment, and *C*_*i*_ be the corresponding censoring time for individual *i*. Since the *j*^*th*^ failure time *T*_*ij*_ is affected by right censoring we define *X*_*ij*_ as the minimum of (*T*_*ij*_,*C*_*i*_), i.e., *X*_*ij*_ equals *T*_*ij*_ if the event was observed, and *C*_*i*_ if it is censored. Let *δ*_*ij*_=*I*(*T*_*ij*_≤*C*_*i*_) be the indicator function taking the value 1 when *T*_*ij*_≤*C*_*i*_ and 0 otherwise. For simplicity, in our simulations all censoring times are taken as constant, i.e. *C*_*i*_=*C* for all *i*.

### Overview of the multiple failure time models

For multiple events the common approaches that generalize the Cox’s framework are the independent increment (AG), marginal (WLW) and conditional (PWP) models. These three models differ essentially in the risk sets.

#### Andersen and Gill (AG) independent increment model

In the AG model [4] it is assumed that recurrent events are unaffected by earlier events that occurred to the same subject so baseline hazards for all events are common. The assumption of mutual independence of the events within a subject is equivalent to the assumption of independent increments in the counting process inside each individual. Each recurrent event for the *i*^*th*^ subject is assumed to follow a proportional hazard model where the hazard function is

$$h_{i}(t)=h_{0}(t)\text{exp}(\beta^{\prime }Z_{i}(t)).$$

Under this model, the risk of a recurrent event for a subject follows the usual proportional hazards assumption but the rank of recurrence is not taken into account. By defining the risk set indicator *Y*_*ij*_(*t*)=*I*(*X*_*i*,*j*−1_<*t*<*X*_*ij*_), the risk set at time *t*, represented by $\underset{\text{ij}}{\sum }Y_{\text{ij}}(t)$, includes all subjects under observation regardless of the number of occurrences experienced by each subject. As *j* increases, fewer individuals are likely to experience the event, but this fact does not affect the risk set due to the fact that it does not depend on *j*; so the coefficients estimates are stable. The AG model provides more efficient inference for a covariate effect than the plain Cox model for the time to the first event, but requires much stronger assumptions than the other models, such as independence among recurrent event times or common baseline hazards. Also, a robust “sandwich” method can be used in the estimation of standard errors [7].

#### Prentice, Williams and Peterson (PWP) conditional model

Another model for analyzing recurrent events is the PWP model [5] with time scale measured from the beginning of the study to a specified failure. It assumes that a subject is not at risk for the *j*^*th*^ event until he/she has experienced event *j*−1. This model requires the same assumptions as the Cox model, but, unlike the AG model, it allows the baseline hazard to vary from recurrence to recurrence, the hazard function for the *j*^*th*^ event for the *i*^*th*^ subject is:

$$h_{\text{ij}}(t)=h_{0j}(t)\text{exp}(\beta_{j}^{\prime }Z_{i}(t)).$$

The risk set indicator is the same as in the AG model, *Y*_*ij*_(*t*)=*I*(*X*_*i*,*j*−1_<*t*<*X*_*ij*_), but the risk set at time *t* is different for each *j*: $\underset{i,}{\sum }Y_{\text{ij}}(t)$. According to the definition of the risk set, the number of subjects is dramatically decreased as *j* increases. Stable coefficient estimates cannot be obtained for higher ranks of *j*. The hazard function at time *t* for the *j*^*th*^ recurrence is conditional to the entire previous failures. This model allows different baseline hazards, therefore, estimations for the current event may be affected by earlier events.

#### Wei, Lin, and Weissfeld (WLW) marginal model

The WLW model [6] uses, for each *j*, the distribution of time from the study enrollment to the *j*^*th*^ recurrent event according to a Cox model, leaving the dependence structure between the observations within the same subject completely unspecified. For the WLW model the risk set indicator is *Y*_*ij*_(*t*)=*I*(*X*_*i*,*j*_≥*t*), and the risk set at time *t* for the *j*^*th*^ recurrence, represented by $\underset{i,}{\sum }Y_{\text{ij}}(t)$, includes anyone who has not experienced the *j*^*th*^ recurrence at time *t*. In other words, unlike the PWP model, a subject in the risk set at time *t* does not necessarily have to experience the (*j*−1)^*th*^ recurrence. The hazard function of the marginal model for the *j*^*th*^ event for the *i*^*th*^ subject is

$$h_{\text{ij}}(t)=h_{0j}(t)\text{exp}(\beta_{j}^{\prime }Z_{i}(t)).$$

The WLW approach obtains the estimate of the covariate effects from the partial likelihood function under the working assumption of independence and then adjusts the variance estimate empirically. Although observations on each subjects are correlated, the *β* estimation has been shown to be consistent in the case of two events [8]. As the inverse of the information matrix provides a naive variance-covariance estimation, not correctly estimating the true variance, a robust “sandwich” variance-covariance estimation method is preferred.

In all these models we can add a frailty, that is, a random individual effect to explain the dependence of individual recurrent events. In our simulation study we try to precisely control the degree of correlation between adjacent recurrent event times. Frailty makes difficult such a control, so we have not considered it.

### Simulation

We carried out a series of simulations to examine the accuracy of the above multiple failure time models in terms of four factors: different sample sizes, censoring levels, numbers of recurrence events and correlation levels. The sample sizes under consideration were *n*=(50,100,200,400). Survival times were censored at fixed time determined to yield censoring percentages *p* of 0%, 15%, 30% and 50%. In other words, previously fixed censoring times *C*_*p*_ = (*C*_0_ = +*∞*,*C*_15_,*C*_30_,*C*_50_) were established in order to ensure the preceding proportions of subjects experiencing a censure. The maximum number of recurrent events under consideration were *K*=(3,6,9,12). For subject *i*, the recurrence times *t*_*i*1_, *t*_*i*2_, …, *t*_*iK*_ were generated as correlated Weibull deviates as is explained below. Censoring occurred in subject *i* if *t*_*i*1_+*t*_*i*2_+…+*t*_*iK*_>*C*_*p*_. In this case he/she experienced *j* events, *j*<*K*, if *T*_*ij*_=*t*_*i*1_+…+*t*_*ij*_≤*C*_*p*_ and *t*_*i*1_+…+*t*_*i*(*j*+1)_>*C*_*p*_. Note that not always censoring occurred only in the *K*^*th*^ event. The correlation levels between adjacent recurrence times were set at *ρ*=(0,0.10,0.45,0.80). The 320 simulated scenarios were defined by crossing all levels of these four factors. Each simulation series consisted of the generation of *m* = 10000 random samples, each one representing a survival dataset where half of individuals were considered as “control” and half of individuals were considered as “treatment”. This covariate was represented by a binary variable with values 0 and 1. The treatment effect was set at *β*=−1. For each simulated dataset we adjusted the WLW, AG and PWP models. We used the following measures of the accuracy of the regression parameter estimates and the gain of robustness from the robust approaches: the true coverage of the 95% confidence interval for the parameter *β*, the relative bias of the *β* estimators under the three models and the bias of the “robust” and “naive” variance estimators for all three models. The relative sampling bias is defined as the average bias from the *m* random samples. That is, if ${\hat{\beta }}_{i}$ is the estimate on *i*^*th*^ simulated dataset, then

$$\begin{array}{ll} \text{relative sampling bias}=\frac{1}{m}\overset{m}{\underset{i=1}{\sum }}\frac{\left({\hat{\beta }}_{i}-\beta \right)}{\beta } & \; \end{array}$$

To simulate the survival times that meet the proportional hazards assumption we used the algorithm proposed by Feiveson [9]. Let *U* be a random variable with uniform distribution. We can generate a new random variable *Y* with distribution function *F* by using the inverse (or pseudo inverse) function *F*^−1^[10] as follows

$$Y=F^{-1}(U).$$

In our case, we are interested in simulating times with Weibull distribution with parameters *c* and *κ*, that is, with distribution function *F*(*y*)=1−*exp*(−*cy*^*κ*^), for *c*>0, *κ*>0. Note that, usually, the Weibull parameters are *λ* and *κ* with *c*=*λ*^*κ*^. Therefore, given U∽ Uniform [0,1],

(2) $$Y={\left(\frac{-\log U}{c}\right)}^{\frac{1}{\kappa }}$$

has the aforementioned Weibull distribution. The hazard function corresponding to *Y* is given by *h*(*y*)=*cκy*^*κ*−1^. Under the Cox model and for a binary covariate *X*, the parameter *c* would be equal to some base value *c*_0_ when *X*=0 and would become equal to *c*_1_=*c*_0_ exp(*β*) when *X*=1.

#### Simulation of within-subject dependence

Our goal is to simulate recurrent Weibull failure times with correlation *ρ* between successive recurrence events in the same individual and with decreasing correlation as the distance between events increases. To incorporate this dependence into our simulated data while still maintaining the marginal Weibull distribution for each observation we will use a transformation of a convenient multivariate normal distribution. We describe this procedure in three steps: 

1. For any correlation *ρ*, choose an appropriate *ρ*_0_ following formula (9) derived in the next section. For each subject, *i*, *i*=1,…,*n*, generate a random variate *Z*_*i*1_ from a *N*(0,1) distribution. Given *z*_*i*1_, generate a random variate *z*_*i*2_ from a normal distribution with mean *ρ*_0_*z*_*i*1_ and variance $1-\rho_{0}^{2}$. Given *z*_*i*2_, generate a random variate *z*_*i*3_ from a $N(\rho_{0}z_{i2},1-\rho_{0}^{2})$; and so on, until *z*_*iK*_. This ensures that each consecutive pair (*z*_*ij*_,*z*_*i*,*j*+1_) is a realization of (*Z*_*ij*_,*Z*_*i*,*j*+1_) following a bivariate normal distribution with standard marginals, *N*(0,1) and correlation *ρ*_0_. Also the correlation between *Z*_*ij*_ and *Z*_*i*,*j*+*τ*_ decreases as *τ* increases.

2. Transform *Z*_*ij*_ into uniform random variables using the standard normal cumulative distribution function *Φ*: *U*_*ij*_=*Φ*(*Z*_*ij*_). Variables *U*_*ij*_ have a similar correlation structure.

3. Finally, transform the variables *U*_*ij*_ into dependent Weibulls using Equation (2). These Weibulls have again a similar correlation structure, with correlation *ρ* between adjacent recurrence times.

#### Computation of the correlation *ρ*_0_

In order to simplify our argumentation we use the logarithm of Weibull times. Using delta method it is easy to see that the correlation between times is approximately equal to the correlation between time logarithms. In terms of the original normally distributed *Z*_*ij*_, the simulated log failure times may be expressed as *g*_*ij*_=*G*(*Z*_*ij*_), where

(3) $$G(z)=\log{\left[\frac{-\log\Phi (z)}{c}\right]}^{\frac{1}{\kappa }}$$

Using second-order Taylor expansions of *g*_*ij*_ and *g*_*i*,*j*+1_ about *z*=0, it can be shown (see Appendix) that

(4) $$\text{Cov}\left(g_{\text{ij}},g_{i,j+1}\right)\approx {\left\{G^{\prime }(0)\right\}}^{2}\rho_{0}+\frac{{\left\{G^{\prime \prime }(0)\right\}}^{2}}{4}\rho_{0}^{2}$$

and

(5) $$\text{Var}\left(g_{\text{ij}}\right)=\text{Var}\left(g_{i,j+1}\right)\approx {\left\{G^{\prime }(0)\right\}}^{2}+\frac{{\left\{G^{\prime \prime }(0)\right\}}^{2}}{2}.$$

Combining (4) and (5), we find that *Corr*(*g*_*ij*_,*g*_*i*,*j*+1_) is a weighted average of *ρ*_0_ and $\rho_{0}^{2}$; namely,

(6) $$\text{Corr}\left(g_{\text{ij}},g_{i,j+1}\right)\approx w\rho_{0}+\frac{1}{2}(1-w)\rho_{0}^{2}$$

where

(7) $$w=\frac{{\left\{G^{\prime }(0)\right\}}^{2}}{{\left\{G^{\prime }(0)\right\}}^{2}+{\left\{G^{\prime \prime }(0)\right\}}^{2}/2}\cdot$$

It can be shown (see Appendix) that *w* does not depend on the parameters *κ* or *c*; specifically,

(8) $$w=\frac{\pi {\left(\log2\right)}^{2}}{\pi {\left(\log2\right)}^{2}+{\left(1-\log2\right)}^{2}}\approx 0.94128$$

Thus, solving (6) for the value of *ρ*_0_ such that *Corr*(*g*_*ij*_,*g*_*i*,*j*+1_)=*ρ*, the solution is

(9) $$\rho_{0}=\frac{-w+\sqrt{w^{2}+2\rho \left(1-w\right)}}{\left(1-w\right)}.$$

#### Software simulation

All the simulations were performed using the library *survival* and the functions *qnorm* and *runif* from the statistical software R2.15.2, 64 bit version, [11]. The default generator is based on a Mersenne-Twister algorithm.

## Results

Table 1 shows results for a simple size of 200 subjects and 15% censoring. For simplicity, we provide only graphical results for two degrees of correlation between adjacent recurrent event times (0.1 and 0.8) and two maximum numbers of possible recurrent events (3 and 9). The complete simulation results corresponding to all 320 simulation scenarios are accessible at the url http://www.ub.edu/stat/recerca/materials/SupplEmpirical.htm.

**Table 1:** Relative bias, naive variance, robust variance and coverage robust for 15% censoring and sample size of 200

***ρ***	**Events**	**Model**	**Relative bias**	**Naive var**	**Robust var**	**Coverage**
0.1	3	AG	-0.193	0.008	0.006	0.308
		PWP	-0.030	0.010	0.011	0.930
		WLW	0.320	0.009	0.021	0.415
	6	AG	-0.254	0.004	0.003	0.003
		PWP	-0.042	0.006	0.007	0.897
		WLW	0.666	0.005	0.021	0.004
	9	AG	-0.278	0.003	0.002	0.000
		PWP	-0.048	0.005	0.006	0.871
		WLW	0.929	0.004	0.022	0.000
	12	AG	-0.291	0.002	0.001	0.000
		PWP	-0.050	0.004	0.005	0.861
		WLW	1.151	0.003	0.022	0.000
0.45	3	AG	-0.296	0.008	0.007	0.069
		PWP	-0.201	0.009	0.011	0.491
		WLW	0.166	0.009	0.022	0.806
	6	AG	-0.343	0.004	0.004	0.001
		PWP	-0.291	0.005	0.006	0.055
		WLW	0.358	0.005	0.022	0.318
	9	AG	-0.350	0.003	0.003	0.000
		PWP	-0.324	0.004	0.004	0.008
		WLW	0.514	0.003	0.021	0.058
	12	AG	-0.349	0.002	0.002	0.000
		PWP	-0.338	0.003	0.003	0.002
		WLW	0.657	0.003	0.022	0.005
0.8	3	AG	-0.381	0.008	0.008	0.016
		PWP	-0.339	0.009	0.011	0.124
		WLW	0.051	0.009	0.024	0.936
	6	AG	-0.448	0.004	0.005	0.000
		PWP	-0.509	0.005	0.005	0.000
		WLW	0.110	0.004	0.023	0.892
	9	AG	-0.462	0.003	0.004	0.000
		PWP	-0.574	0.003	0.003	0.000
		WLW	0.157	0.003	0.023	0.829
	12	AG	-0.462	0.002	0.004	0.000
		PWP	-0.605	0.002	0.003	0.000
		WLW	0.205	0.002	0.022	0.727

If only the first event is considered, the relative bias, efficiency and coverage of the standard Cox proportional hazard estimator are adequate, as expected, in all the investigated scenarios. We do not report these results because our interest is focused in recurrent data.

Censoring has a very small influence on the performance of the treatment effect estimators, $\hat{\beta }$, for all models under consideration. The graphics are nearly identical for all levels of censoring so only 15% level is reported.

**Figure 1:**
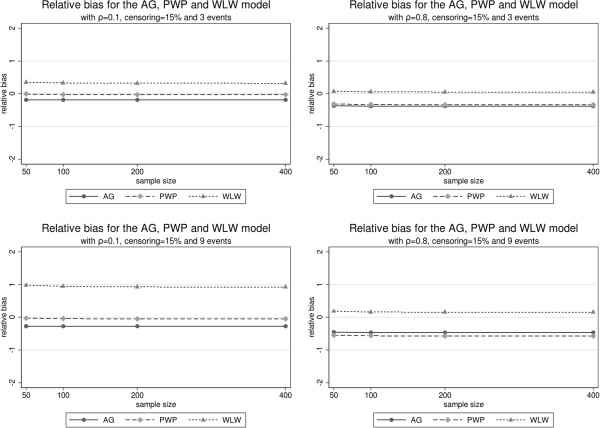
Relative Bias for each model with correlation of 0.1 and 0.8, 15% censoring and 3 and 9 events.

**Figure 2:**
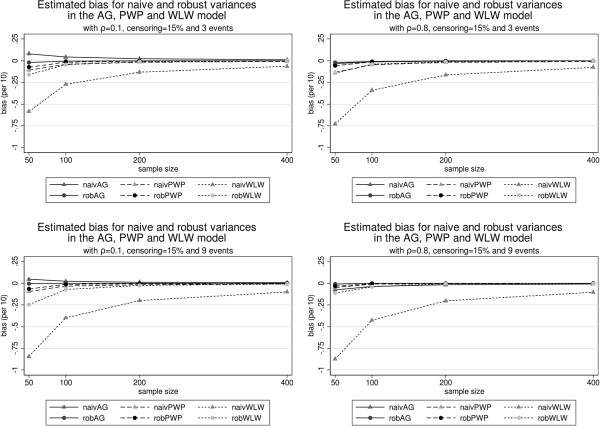
Estimated Bias for naive and robust variances for each model with correlation of 0.1 and 0.8, 15% censoring and 3 and 9 events.

**Figure 3:**
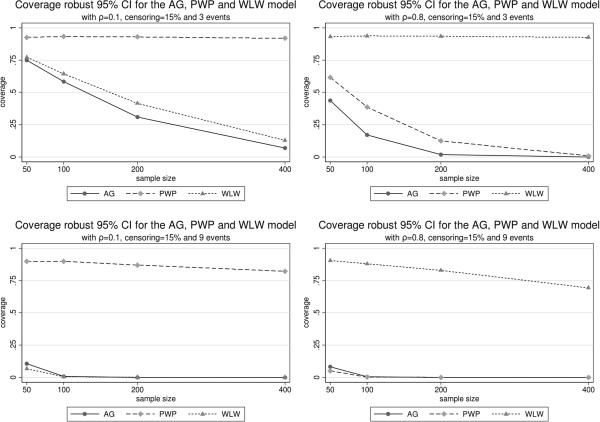
Coverage robust 95% confidence intervals for each model with correlation of 0.1 and 0.8, 15% censoring and 3 and 9 events.

Figure 1 illustrates the relative bias, Figure 2 the bias of the variance estimators and Figure 3 the true coverage of the 95% confidence interval based on the robust variance estimator. All these figures are organized in rows and columns. Each column corresponds to a correlation level (*ρ*=0.1 and *ρ*=0.8) and each row to a maximum number of possible recurrences (*K*=3 and *K*=9).

With respect to relative bias, the AG estimator of *β* is stable for all different sample sizes. The bias grows with the correlation and the number of possible recurrences. The treatment effect is always underestimated. In all circumstances the naive variance estimator is worse than the robust estimator, but both perform very similarly and adequately. The true coverage of the confidence intervals is in general low and decreases with correlation, number of events and, surprisingly, with sample size.

The estimator of *β* based on model PWP is nearly unbiased for low correlations and stable with respect to the number of recurrences. When the correlation is high, it underestimates the treatment effect *β*. This bias does not improve with growing sample sizes. Again, the robust variance is better than the naive variance, but both are very similar and perform adequately, improving with growing sample sizes and correlations. For low correlation levels its coverage is the best, but it quickly falls with increasing correlations and becomes sensitive to growing sample sizes, in the same surprising direction as the AG method.

Under the WLW model *β* is overestimated. This bias increases with growing recurrence but improves with growing correlation. The naive variance estimator under WLW is very biased, its use should be avoided. The robust variance estimator is clearly better but slightly worse than the other robust estimators for sample sizes below 200. Under low correlation, the confidence interval performs very inadequately, with very low coverages which decrease with growing sample sizes and with growing number of recurrences. On the other hand, its coverage greatly improves with growing correlations, though a tendency to make worse with growing sample sizes and recurrence levels still persists.

### Example

The models under consideration are illustrated through the analysis of a cohort of infants with bronchial obstruction [12]. For this study, 4 months-old children were followed until they reached 12 months of age and recruitment took place during the course of 18 months, from April 1995 to October 1996. The chief goal of the investigation was to estimate the effects of fine particulate matter and wheezing illness in the first year of life. The recurrent events of interest were physician office visits attributable to respiratory illnesses. From the pediatric visit database, a total of 504 infants were eligible for the present study in the pediatric visit database. The events of interest were the times to physician revisit following the initial visit due to examination. Each subject experienced a particular number of visits to physician at various times, which represent the whole observable history of his/her recurrences. For each individual the last time is censored due to the end of the study period. Of these 504 subjects, 475 had at least one revisit during the follow-up time. Table 2 summarizes the number of events experienced by the 475 subjects during the follow-up time. A total of 475 occurrences on revisit were observed, where some infants experienced a rather large number (up to 10 revisits).

**Table 2:** Number of episodes of illness

	**Number of events**							
	**0**	**1**	**2**	**3**	**4**	**5**	**6-10**	**Total**
N. of subjects (%)	260 (54.7)	101 (21.3)	53 (11.2)	21 (4.4)	21 (4.4)	10 (2.1)	9 (1.9)	475 (100)

The main purpose of this example is to illustrate the application of recurrent event techniques and provide comparisons between techniques, rather than giving estimates for recurrence of respiratory illnesses. Two characteristics, parents reported smoking (1=yes, 0=no) and family history of asthma (1=yes, 0=no) were included as two univariate analysis in each of the models studied. We chose these two covariates because they are potentially related with the outcome as indicated in the medical literature and also because they are binary variables as we used in our simulation study. Table 3 contains the results of three different models: AG, PWP and WLW, fitted to the respiratory data. The *β* values estimated by the AG and PWP models are lower than those estimated by the WLW model for both covariates, smoking and asthma. But all of them show a negative effect in the outcome. When we analyze the standard errors, the robust standard errors are larger in all models than those estimated by the naive method. And again, the WLW model has the biggest standard error in comparison to the other models. These results are coherent with the previous simulation results. Note that the WLW is the only model where asthma is a statistically significant.

**Table 3:** Coefficients and standard errors obtained in two univariate analysis for 2 covariates: parents reported smoking and family history of asthma

**Model**	**Covariate**	**Beta**	**Naive S.E.**	**Robust S.E.**	**Robust 95% CI**	
AG	Smoking	0.158	0.059	0.089	-0.016, 0.332	
	Asthma	0.276	0.111	0.155	-0.028, 0.579	
PWP	Smoking	0.147	0.060	0.088	-0.025, 0.319	
	Asthma	0.251	0.113	0.148	-0.039, 0.540	
WLW	Smoking	0.162	0.058	0.102	-0.037, 0.362	
	Asthma	0.407	0.111	0.189	0.036, 0.778	

## Discussion

The simulation results show that each model has different degrees of robustness against variations in the number of recurrent events and correlation. In terms of relative bias, the WLW model shows the worst performance for low correlations but greatly improves for high correlation levels, independently of censoring and sample size. The situation is reversed for the AG and PWP models, which perform better than WLW for low correlation levels. However, these models clearly underestimate the parameter. As some authors have suggested [3], the WLW model overestimates regression coefficients due to a carry-over effect. This is corroborated by our simulation results. Additionally, as we can see from Table 3 in the example, the WLW model presents the highest coefficients and robust standard errors.

The most surprising results refer to the unexpected behavior of all methods with growing sample sizes. First, their bias remains nearly constant. This was so surprising that we repeated the computations with Stata, with identical results to those obtained with the “coxph” R function. Provided that (as is expected) the true variance of all estimators decreases and its robust estimation greatly improves with growing sample sizes (Figure 2), as a consequence the confidence intervals become more and more narrow around a biased value, which produces a disconcerting, at first sight, fall of coverage (Figure 3).

The AG, PWP and WLW models have been previously compared by various authors (e.g., [3,7,13,14]) using real and simulated data, showing that these models often yield different results for the same data set. This is not unexpected, since distinct models address different research questions. For example, in [3] they found that the WLW model overestimates the treatment effect. Also they show that as the correlation increases the coverage of the 95% confidence interval based on the robust variance decreases, except for WLW.

## Conclusion

In this paper we reviewed and compared the principal methods in the analysis of recurrent event data in terms of censoring and within-subject correlation. The example illustrates the differences between the methods and resulting parameter estimators.

No method can be recommended as the best in all circumstances. The AG and PWP approaches are quite robust in front of low levels of within-subject correlation, but the WLW approach should be recommended under the suspicion of high correlation. All methods share a bias problem which makes the confidence intervals based on asymptotic normal theory not fully accurate. On the other hand, the robust estimation of the variance of the treatment effect estimator is very reliable, and should be recommended instead of the naive variance estimation, especially for the WLW method.

## Appendix

Remember that *ρ*(*Z*_*ij*_,*Z*_*ik*_)=*ρ*_0_>0 and *ρ*(*g*_*ij*_,*g*_*i*,*j*+1_)=*ρ*>0 with *g*_*ij*_=*G*(*Z*_*ij*_) where *G* is defined in (3). To find the correlation between *g*_*ij*_ and *g*_*i*,*j*+1_ we will use a second order Taylor expansion (delta method, [15]) for the function *G* about *z*=0 as follows:

$$\begin{array}{lll} \;g_{\text{ij}}\;=\;G(Z_{\text{ij}}) & =G(0)\;+\;G^{\prime }(0)(Z_{\text{ij}}-0)\;+\;\frac{G^{\prime \prime }(0)}{2}{\left(Z_{\text{ij}}\;-\;0\right)}^{2}\;+\;\cdots \; & \;\\ & \approx G(0)+G^{\prime }(0)Z_{\text{ij}}+\frac{G^{\prime \prime }(0)}{2}Z_{\text{ij}}^{2}\; & \; \end{array}$$

since *Z*_*ij*_∼*N*(0,1), we have *E*(*Z*_*ij*_)=0 and $\text{Var}(Z_{\text{ij}})=E(Z_{\text{ij}}^{2})=1$, then,

$$\begin{array}{lll} E(g_{\text{ij}}) & \approx G(0)+\frac{G^{\prime \prime }(0)}{2}\; & \;\\ g_{\text{ij}}-E(g_{\text{ij}}) & \approx G^{\prime }(0)Z_{\text{ij}}+\frac{G^{\prime \prime }(0)}{2}(Z_{\text{ij}}^{2}-1)\; & \;\\ g_{i,j+1}-E(g_{i,j+1}) & \approx G^{\prime }(0)Z_{\text{ik}}+\frac{G^{\prime \prime }(0)}{2}(Z_{\text{ik}}^{2}-1)\; & \; \end{array}$$

and

$$\begin{array}{lll} \;(g_{\text{ij}} & -E(g_{\text{ij}}))(g_{i,j+1}-E(g_{i,j+1}))\approx {\left(G^{\prime }(0)\right)}^{2}Z_{\text{ij}}Z_{\text{ik}}\; & \;\\ & +\frac{G^{\prime }(0)G^{\prime \prime }(0)}{2}Z_{\text{ij}}(Z_{\text{ik}}^{2}-1)+\frac{G^{\prime }(0)G^{\prime \prime }(0)}{2}Z_{\text{ik}}(Z_{\text{ij}}^{2}-1)\; & \;\\ & +\frac{{\left(G^{\prime \prime }(0)\right)}^{2}}{4}(Z_{\text{ij}}^{2}-1)(Z_{\text{ik}}^{2}-1).\; & \; \end{array}$$

By applying now the *E*(·) operator taking into account that

$$(Z_{\text{ij}},Z_{\text{ik}})∼N\left(\left(\begin{array}{l} 0\\ 0 \end{array}\right),\left(\begin{array}{ll} 1 & \rho_{0}\\ \rho_{0} & 1 \end{array}\right)\right),$$

so *E*[*Z*_*ij*_*Z*_*ik*_]=*ρ*_0_ and $E[Z_{\text{ij}}(Z_{\text{ik}}^{2}-1)]=E[Z_{\text{ik}}Z_{\text{ij}}^{2}]=0$, we have:

$$\begin{array}{lll} \text{Cov}(g_{\text{ij}},g_{i,j+1}) & \approx {\left(G^{\prime }(0)\right)}^{2}E(Z_{\text{ij}}Z_{\text{ik}})+\frac{{\left(G^{\prime \prime }(0)\right)}^{2}}{4}\; & \;\\ & \;\times E\left[\left(Z_{\text{ij}}^{2}-1)(Z_{\text{ik}}^{2}-1\right)\right]\; & \;\\ & ={\left(G^{\prime }(0)\right)}^{2}\rho_{0}+\frac{{\left(G^{\prime \prime }(0)\right)}^{2}}{4}\rho_{0}^{2}.\; & \; \end{array}$$

For the *Var*(*g*_*ij*_) we proceed in the same way as before,

$$\begin{array}{lll} \text{Var}(g_{\text{ij}}) & \approx \text{Var}\left(G(0)+G^{\prime }(0)Z_{\text{ij}}+\frac{G^{\prime \prime }(0)}{2}Z_{\text{ij}}^{2}\right)\; & \;\\ & ={\left(G^{\prime }(0)\right)}^{2}\text{Var}(Z_{\text{ij}})+\frac{1}{4}{\left(G^{\prime \prime }(0)\right)}^{2}\text{Var}(Z_{\text{ij}}^{2})\; & \;\\ & ={\left(G^{\prime }(0)\right)}^{2}+\frac{1}{2}{\left(G^{\prime \prime }(0)\right)}^{2}.\; & \; \end{array}$$

because $\text{Cov}(Z_{\text{ij}},Z_{\text{ik}}^{2})=0$, and $\text{Var}(Z_{\text{ij}}^{2})=2$. Finally the formula for the approximated correlation between *g*_*ij*_ and *g*_*i*,*j*+1_ is

$$\begin{array}{lll} \text{Corr}(g_{\text{ij}},g_{i,j+1}) & =\frac{\text{Cov}(g_{\text{ij}},g_{i,j+1})}{\sqrt{\text{Var}(g_{\text{ij}})\text{Var}(g_{i,j+1})}}\; & \;\\ & =\frac{{\left(G^{\prime }(0)\right)}^{2}\rho_{0}+\frac{1}{4}{\left(G^{\prime \prime }(0)\right)}^{2}\rho_{0}^{2}}{{\left(G^{\prime }(0)\right)}^{2}+\frac{1}{2}{\left(G^{\prime \prime }(0)\right)}^{2}}\; & \;\\ & =\frac{{\left(G^{\prime }(0)\right)}^{2}}{{\left(G^{\prime }(0)\right)}^{2}+\frac{1}{2}{\left(G^{\prime \prime }(0)\right)}^{2}}\rho_{0}\; & \;\\ & \;\;+\frac{1}{4}\frac{{\left(G^{\prime \prime }(0)\right)}^{2}}{{\left(G^{\prime }(0)\right)}^{2}+\frac{1}{2}{\left(G^{\prime \prime }(0)\right)}^{2}}\rho_{0}^{2}\; & \;\\ & =w\cdot \rho_{0}+\frac{\left(1-w\right)}{2}\rho_{0}^{2}\; & \; \end{array}$$

where, $w=\frac{{\left(G^{\prime }(0)\right)}^{2}}{{\left(G^{\prime }(0)\right)}^{2}+\frac{1}{2}{\left(G^{\prime \prime }(0)\right)}^{2}}\cdot$ In order to compute *w*, we use that:

(10) $$\begin{array}{ll} \Phi (z)=\overset{z}{\underset{-\infty }{\int }}\frac{1}{\sqrt{2\pi }}e^{-\frac{x^{2}}{2}}\text{dx} & \;\Phi (0)=\frac{1}{2}\\ \Phi^{\prime }(x)=\frac{1}{\sqrt{2\pi }}e^{-\frac{x^{2}}{2}} & \;\Phi^{\prime }(0)=\frac{1}{\sqrt{2\pi }}\\ \Phi^{\prime \prime }(x)=-\frac{x}{\sqrt{2\pi }}e^{-\frac{x^{2}}{2}} & \;\Phi^{\prime \prime }(0)=0. \end{array}$$

Then,

$$\begin{array}{lll} G^{\prime }(z) & = & \frac{1}{k}\frac{\Phi^{\prime }(z)}{\Phi (z)\log\Phi (z)}\\ G^{\prime }(0) & = & -\frac{\sqrt{2}}{k(\log2)\sqrt{\pi }}\\ G^{\prime \prime }(z) & = & -\frac{1}{k}\frac{{\left(\Phi^{\prime }(z)\right)}^{2}}{{\left(\log\Phi (z)\right)}^{2}\Phi {\left(z\right)}^{2}}+\frac{1}{k}\frac{\Phi^{\prime \prime }(z)\Phi (z)-{\left(\Phi^{\prime }(z)\right)}^{2}}{\Phi {\left(z\right)}^{2}\log\Phi (z)}\\ G^{\prime \prime }(0) & = & \frac{2(\log2-1)}{\mathrm{k\pi}{\left(\log2\right)}^{2}}. \end{array}$$

And finally,

$$\;w=\frac{{\left(G^{\prime }(0)\right)}^{2}}{{\left(G^{\prime }(0)\right)}^{2}+\frac{1}{2}{\left(G^{\prime \prime }(0)\right)}^{2}}=\frac{\pi {\left(\log2\right)}^{2}}{\pi {\left(\log2\right)}^{2}+{\left(\log2-1\right)}^{2}}.$$

Note that *w* does not depend neither on *k* nor on *c*.

## Competing interests

The authors declare that they have no competing interests.

## Authors’ contributions

RV conducted literature review, wrote computer programs, produced graphs and contributed to the mathematical development. OJ developed the mathematical framework and derived mathematical results. JO contributed to the improvement of simulation techniques and software development. All authors prepared the manuscript and have read and approved the final version.

## Pre-publication history

The pre-publication history for this paper can be accessed here:

http://www.biomedcentral.com/1471-2288/13/95/prepub
